# In vitro additive antitumor effects of dimethoxycurcumin and 5‐fluorouracil in colon cancer cells

**DOI:** 10.1002/cam4.1114

**Published:** 2017-06-02

**Authors:** Huiying Zhao, Qingchun Liu, Saisai Wang, Fang Dai, Xiaofei Cheng, Xiaobin Cheng, Wenbin Chen, Min Zhang, Dong Chen

**Affiliations:** ^1^ Department of Colorectal Surgery The First Affiliated Hospital College of Medicine Zhejiang University Hangzhou 310003 China; ^2^ Department of Colorectal Surgery Yiwu Traditional Chinese Medicine Hospital Yiwu 322000 China; ^3^ State Key Laboratory of Applied Organic Chemistry Lanzhou University Lanzhou 730000 China; ^4^ Key Laboratory of Precision Diagnosis and Treatment for Hepatobiliary and Pancreatic Tumor of Zhejiang Province Hangzhou Zhejiang China; ^5^ College of Medicine Zhejiang University 866 Yuhangtang Road Hangzhou 310058 Zhejiang China

**Keywords:** 5‐fluorouracil (5‐Fu), additive antitumor effect, colon cancer, dimethoxycurcumin

## Abstract

Dimethoxycurcumin (DMC) is a lipophilic analog of curcumin, an effective treatment for colon cancer, which has greater chemical and metabolic stability. Chemotherapy treatments, such as 5‐fluorouracil (5‐Fu), play a key role in the current management of colon cancer. In this study, we investigated the antitumor efficacy of DMC in combination with 5‐Fu in SW480 and SW620 colon cancer cells. CCK‐8 assay was used to evaluate the inhibitory effect of DMC and 5‐Fu on cancer cells proliferation, and the combination index was calculated. The influence of DMC and 5‐Fu on cell cycle, apoptosis, reactive oxygen species (ROS) production, and mitochondrial membrane potential in SW480 and SW620 cells was determined using flow cytometry, and the related signaling pathways were detected by western blot. Transmission electron microscopy was used to observe endoplasmic reticulum expansion. DMC‐ and/or 5‐Fu‐induced apoptosis, stimulated G0/G1 phase arrest, increased ROS levels, decreased mitochondrial membrane potential, and enhanced endoplasmic reticulum expansion. The induction of apoptosis is involved in the increasing of Bax and cytochrome c and decreasing of Bcl2 expressions. Increased production of ROS was accompanied by upregulation of CHOP and Noxa. Combination therapy of DMC and 5‐Fu had increased efficacy on the above pathways compared with either drug alone. Based on the calculated IC_50_, combination treatment with DMC and 5‐Fu had an additive antitumor effect in both cell lines. Combined treatment with DMC and 5‐Fu led to an additive antitumor effect in colon cancer cells that was related to apoptosis induction, G0/G1 phase arrest, increased ROS production, decreased mitochondrial membrane potential, and enhanced endoplasmic reticulum expansion.

## Introduction

Colon cancer is one of the most common cancers worldwide, with 1.6 million patients diagnosed in 2013 1. In addition, there has been a significant increase in incidence in young people in recent years 2. While the incidence and mortality rates of colorectal cancer in China are lower than the global averages, the incidence trends over the last 20 years have not been positive 3. These trends may be partly due to dietary changes, including a reduced intake of fruits and vegetables 2. Chemotherapy plays a key role in the current management of colon cancer, especially for late‐stage tumors. However, there is great variability in the clinical response to chemotherapy, and cancer cells often develop resistance to treatments, resulting in tumor recurrence and further progression 4. Reducing the effective dose of chemotherapeutic drugs using combination therapies could result in fewer adverse events, therefore, multicomponent therapeutics using several compounds that interact with diverse targets has become a renewed research focus 5.

A large number of phytochemicals with antitumor effects have been discovered, and curcumin is one of the most effective 6. However, low water solubility, metabolic instability, and poor bioavailability limit the clinical application of curcumin 7. Dimethoxycurcumin (DMC), a synthesized lipophilic analog of curcumin, has better chemical and metabolic stability compared with curcumin 8, 9. Moreover, DMC is a potential candidate for the treatment of colon cancer, since it has increased potential to induce apoptosis in colon cancer cells, is less toxic to normal cells, and has a higher bioactivity compared with curcumin 8, 10. A preliminary study from our lab showed that DMC is an effective antitumor agent that downregulates survivin and upregulates E‐cadherin in colon cancer cells 11. Moreover, recent studies reported that curcumin combined with FOLFOX chemotherapy was clinically safe and tolerable and capable of preventing colon cancer growth 12, 13, 14. Therefore, combining DMC with chemotherapy should have potent antitumor effects in colon cancer cells.

Fluoropyrimidines, such as 5‐fluorouracil (5‐Fu) and capecitabine, are the standard chemotherapy treatment for colon cancer 15, 16. However, the response rates are only 10–15% as a result of their severe side effects and resistance 17. Therefore, we examined the antitumor efficacy of DMC in combination with 5‐Fu in SW480 and SW620 cells, which are two representative colon cancer cell lines with distinct phenotypic differences.

## Materials and Methods

### Cell culture and reagents

The colon cancer cell lines SW480 and SW620, provided by the Department of Pathology, College of Medicine, Zhejiang University (Hangzhou, China), were cultured in RPMI 1640 with 10% heat‐inactivated fetal bovine serum, 2 mmol/L glutamine, 50 U/mL penicillin, and 50 *μ*g/mL streptomycin at 37°C in a 5% CO_2_ incubator. All cell culture reagents were purchased from Invitrogen (Shanghai, China).

DMC was supplied by the Department of Applied Chemistry, Lanzhou University (Lanzhou, China). DMC purity was > 90% as detected by high‐performance liquid chromatography. DMC was prepared as a stock solution in dimethyl sulfoxide (DMSO) and stored at −20°C. For each experiment, the stock solutions were diluted with cell culture medium to a final DMSO concentration of 0.5% (v/v).

### CCK‐8 assay

SW480 and SW620 cells were seeded into 96‐well plates at a density of 5 × 103/well and allowed to grow overnight. Cells were then incubated with DMC (0, 1, 4, 8, 16, 32, 64, or 128 *μ*mol/L) or 5‐Fu (0, 1, 4, 8, 16, 32, 64, or 128 mg/L) for 48 h. After incubation, 10 *μ*L CCK‐8 (Beyotime Biotechnology, Jiangsu, China) solution were added to each well. The optical density (OD) was measured at 490 nm using an ultramicroplate reader (SpectraMax; Molecular Devices Co., Sunnyvale, CA). Relative cell growth inhibition was calculated using the following formula: (OD_490_ control cells–D_490_ treated cells)/OD_490_ control cells × 100%. The half‐maximal inhibitory concentration (IC_50_) was defined as the concentration of DMC that produced 50% inhibition of cell growth in comparison with control cell cultures. Experiments were independently repeated three times.

Based on the IC_50_ determined by CCK‐8 assay, we treated SW480 and SW620 cells with 40 or 25 mg/L 5‐Fu and/or 130 or 30 *μ*mol/L DMC, respectively. The concentrations selected were below the IC_50_. We set up four groups for the study: group A (SW480 or SW620 cells), group B (SW480 or SW620 cells + 5‐Fu), group C (SW480 or SW620 cells + DMC), and group D (SW480 or SW620 cells + 5‐Fu + DMC).

### Calculation of the combination index

To determine the combined effects of DMC and 5‐Fu on colon cancer cells, the combination index was calculated. Cells were treated with 5‐Fu and/or DMC at the indicated concentrations for 48 h before performing a CCK‐8 assay. Calculation of the combination index was performed using the following formula: Q = E_a+b_/(*E*_a_ + *E*_b_ −*E*_a_ × *E*_b_), where Q is the combination index; *E*_a + b_ represents the rate of cell proliferation inhibition induced by the combination drug; *E*_a_ and *E*_b_ represent the rate of cell proliferation inhibition of each drug 5. Resulting values of Q < 0.85, Q > 1.15, and 0.85 < Q < 1.15 indicate antagonism, synergy, and an additive effect, respectively.

### Cell cycle analysis

The effect of DMC and/or 5‐Fu on the cell cycle was determined by flow cytometry using a Cell Cycle Analysis kit (Beyotime). In brief, cells were treated with DMC and/or 5‐Fu for 48 h, harvested, washed with PBS twice, and then fixed with 70% ethanol at 4°C for at least 1 h. Cells were stained with 50 mg/ml propidium iodide (PI) in the dark for 30 min and resuspended in PBS at 4°C followed by flow cytometric analysis using CyFlow^®^ SL (PARTEC Company, Germany).

### Apoptosis detection

The effect of DMC and/or 5‐Fu on cell apoptosis was determined by flow cytometry using the Annexin V‐FITC/PI Apoptosis Detection kit (Beyotime). In brief, cells were collected after treatment for 48 h with DMC and/or 5‐Fu, washed with PBS, resuspended in binding buffer, and then incubated with Annexin V‐FITC/PI for 15 min in the dark prior to flow cytometry analysis.

### Measurement of intracellular ROS levels

Accumulation of intracellular ROS was determined by flow cytometry using the Reactive Oxygen Species Assay Kit (Beyotime) as described previously 18. In brief, colon cancer cells were collected after treatment with DMC and/or 5‐Fu and resuspended in serum‐free medium containing 10 *μ*mol/L 2′, 7′‐dichlorodihydrofluorescein. The cells were then incubated at 37 °C for 20 min in the dark with rocking every 5 min. DCF fluorescence intensity was then detected by flow cytometry at an excitation of 488 nm and emission of 525 nm.

### Measurement of mitochondrial membrane potential

A mitochondrial membrane potential assay kit with JC‐1 (Beyotime) was used to detect the mitochondrial membrane potential in colon cancer cells as described previously 19. In brief, cells were incubated with JC‐1 working solution for 20 min at 37°C in the dark after treatment with DMC and/or 5‐Fu for 48 h and then washed twice with ice‐cold JC‐1 buffer solution before analyzing by flow cytometry.

### Western blotting

Cell proteins were extracted using whole cell extraction buffer. Equal amounts of protein were separated by sodium dodecyl sulfate polyacrylamide gel electrophoresis and transferred to polyvinylidene fluoride membranes (Millipore, Germany). Immunoblotting was then performed using monoclonal antibodies against Bax (50599‐2‐lg, Proteintech Group, Inc., Bcl‐2 (12789‐1‐ap, Proteintech Group, Inc.), cytochrome c (Cyt c) (AF0146, Affinity Biosciences, OH), CHOP (DF6025, Affinity Biosciences, OH), and Noxa (17418‐1‐ap, Proteintech Group, Inc.). Each experiment was repeated independently three times.

### Transmission electron microscopy

Transmission electron microscopy was used to observe endoplasmic reticulum expansion in both cell lines. Cells were fixed in Karnovsky's solution (1% paraformaldehyde, 2% glutaraldehyde, 2 mmol/L calcium chloride, 0.1 mol/L cacodylate buffer, pH 7.4) for 2 h and washed with cacodylate buffer. Postfixing was performed in 1% osmium tetroxide and 1.5% potassium ferrocyanide for 1 h. After dehydration with 50–100% alcohol, the cells were embedded in Poly/Bed 812 resin (Pelco, Redding, CA), polymerized, and observed under an electron microscope (EM 902A, Zeiss).

### Statistical analysis

SPSS 19.0 software package (SPSS Inc., Chicago, IL) was used for all analyses. All quantitative variants are expressed as means ± standard deviation (SD). Student's *t* test was used to determine differences between two groups, and the least significant difference method, a type of one‐way ANOVA, was used to determine the differences among three or more groups. The IC_50_ value was calculated using the “Analysis/Regression/Probit” application based on data from the CCK‐8 assay. A value of *P* < 0.05 was considered statistically significant. The results were visualized using GraphPad prism 6 software.

## Results

### DMC and 5‐Fu inhibit colon cancer cells growth in vitro

**Figure 1:**
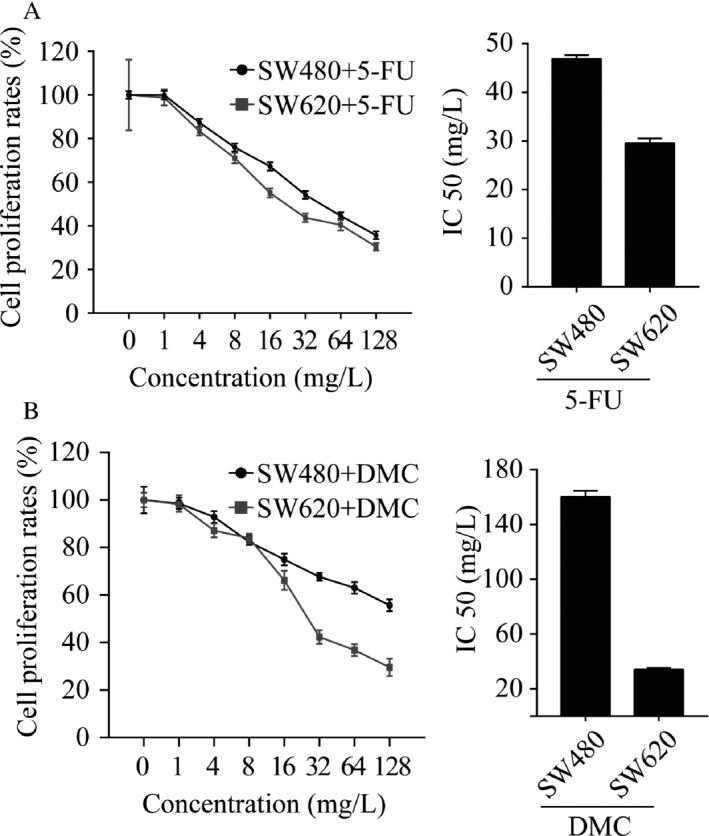
The inhibitory effects of dimethoxycurcumin (DMC) and 5‐fluorouracil (5‐Fu) on colorectal cancer cells growth. (A) CCK‐8 assays were used to analyze the effect of different concentrations of 5‐Fu on SW480 and SW620 cells after treatment for 48 h. (B) CCK‐8 assays were used to analyze the effect of different concentrations of DMC on SW480 and SW620 cells after 48 h of treatment. Results were obtained from three independent experiments and expressed as the means ± SD.

Both DMC and 5‐Fu significantly inhibited the growth of SW480 and SW620 cells in a dose‐dependent manner, and the maximum effect was observed at concentrations of 128 *μ*mol/L and 128 mg/L, respectively. As shown in Figure 1, the IC_50_ for DMC was 160.10 *μ*mol/L for SW480 cells and 34.00 *μ*mol/L for SW620 cells. The IC_50_ for 5‐Fu was 46.88 mg/L for SW480 cells and 29.58 mg/L for SW620 cells. The above results demonstrated that both DMC and 5‐Fu suppressed the growth of colon cancer cells.

### Combination index calculation

**Figure 2:**
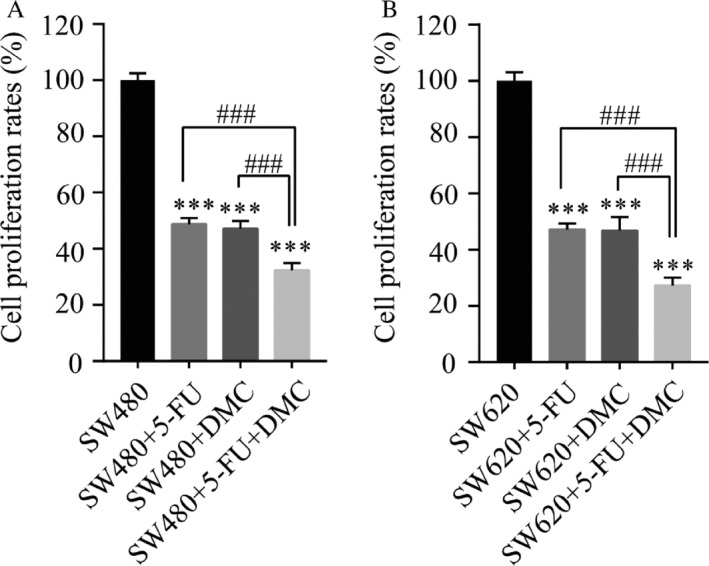
SW480 (A) and SW620 (B) cells viability after treatment with 5‐Fu and/or DMC. Results were obtained from three independent experiments and expressed as the means ± SD. Compared with SW480 (or SW620), ***P < 0.001; compared with SW480 (or SW620) + 5‐Fu + DMC, ^###^ *P*<0.001.

In both SW480 and SW620 cell lines, the inhibition of proliferation rates in groups B (51.28 ± 2.22% and 52.67 ± 2.11%, respectively), C (52.87 ± 2.72% and 53.14 ± 4.77%, respectively), and D (67.65 ± 2.53% and 72.67 ± 2.82%, respectively) were significantly enhanced compared with those in group A. Moreover, the inhibition rate in group D was significantly greater than that in group B or C (Fig. 2). The combination indexes in SW480 and SW620 cells were 0.89 and 0.93, respectively, suggesting that DMC and 5‐Fu exerted an additive antitumor effect in both cell lines.

### Changes in the cell cycle, apoptosis, ROS levels, and mitochondrial membrane potential

**Figure 3:**
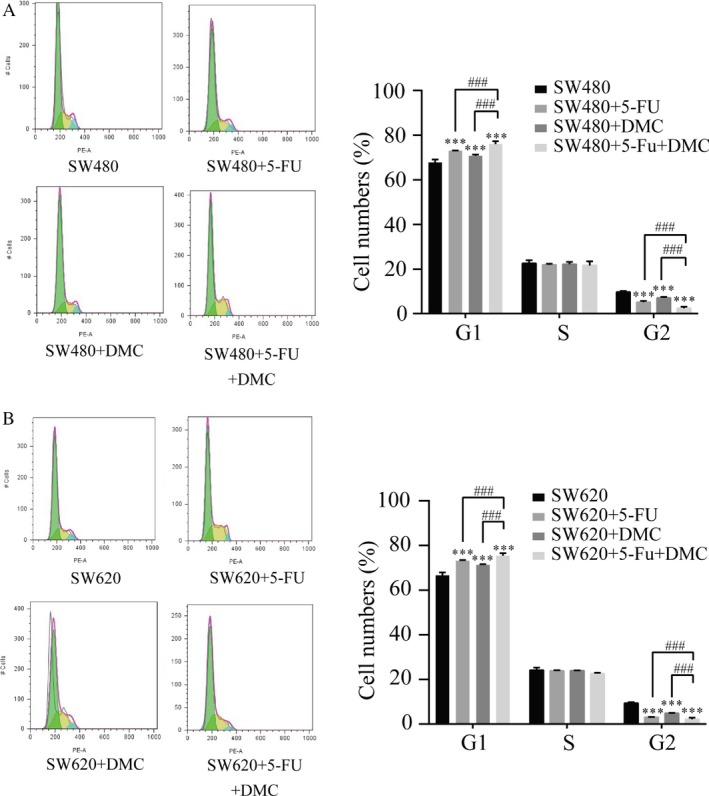
The effects of DMC and/or 5‐Fu on the cell cycle. Cell cycle distribution was examined by PI staining and flow cytometry in both SW480 (A) and SW620 (B) cell lines after treatment with 5‐Fu and/or DMC for 48 h. DMC and 5‐Fu treatment increased the proportion of cells in G1 phase and decreased the proportion of cells in G2 phase in both cell lines. Combination of the two drugs increased these effects. Results were obtained from three independent experiments and expressed as the means ± SD. Compared with SW480 (or SW620), ***P < 0.001; compared with SW480 (or SW620) + 5‐Fu + DMC, ^###^ *P*<0.001

Cell cycle distribution was examined by PI staining and flow cytometry. Results are presented as the representative experiment or the mean values ± SD of three experiments. The proportions of SW480 and SW620 cells in G1 phase showed significant increases in groups B (72. 97 ± 0.16% and 73.13 ± 0.29%, respectively), C (70.73 ± 0.60% and 71.32 ± 0.26%, respectively), and D (76.01 ± 1.24% and 75.35 ± 1.12%, respectively) compared with group A (67.69 ± 1.43% and 66.59 ± 1.33%, respectively). A significant decrease in the proportion of SW480 and SW620 cells in G2 phase was also detected in groups B (5.10 ± 0.61% and 3.04 ± 0.12%, respectively), C (7.13 ± 0.44% and 4.81 ± 0.19%, respectively), and D (2.40 ± 0.67% and 2.10 ± 0.76%, respectively) compared with group A (9.73 ± 0.47% and 9.30 ± 0.48%, respectively). In addition, the proportion of cells in G1 and G2 were further increased and decreased, respectively, in group D compared with groups B and C. The above results were shown in Figure 3.

**Figure 4:**
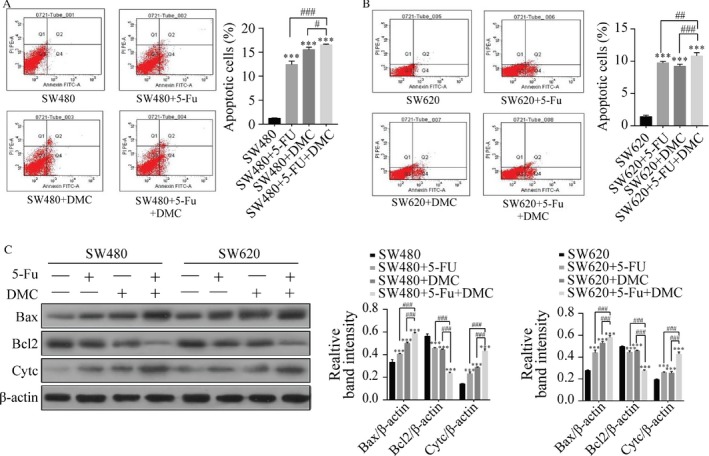
The effects of 5‐Fu and/or DMC on cell apoptosis and apoptotic protein expressions. Annexin V‐FITC/PI was used to detect cell apoptosis in SW480 (A) and SW620 cells (B). The absolute intensity values of Bax, Bcl2, and Cyt c were significantly different in both cell lines after treatment (C). Results were obtained from three independent experiments and expressed as the means ± SD. Compared with SW480 (or SW620), **P < 0.01, ***P < 0.001; compared with SW480 (or SW620) + 5‐Fu + DMC, ^##^ *P* < 0.01, ^###^ *P* < 0.001

Apoptosis was examined by flow cytometry. The percentages of apoptotic SW480 and SW620 cells were significantly increased in groups B (12.43 ± 0.70% and 9.77 ± 0.21%, respectively), C (15.53 ± 0.49% and 9.23 ± 0.32%, respectively), and D (16.57 ± 0.12% and 10.83 ± 0.50%, respectively) compared with group A (1.23 ± 0.12% and 1.43 ± 0.21%, respectively). In addition, the percentage of apoptotic cells in group D was significantly higher than that in group B or C. Protein expression of Bax and Cyt c was increased and that of Bcl2 decreased by DMC and 5‐Fu treatment in both SW480 and SW620 cells in groups B, C, and D compared with group A. These effects were increased in group D compared with groups B and C. The results are shown as the representative experiment or the means ± SD of three experiments in Figure 4.

**Figure 5:**
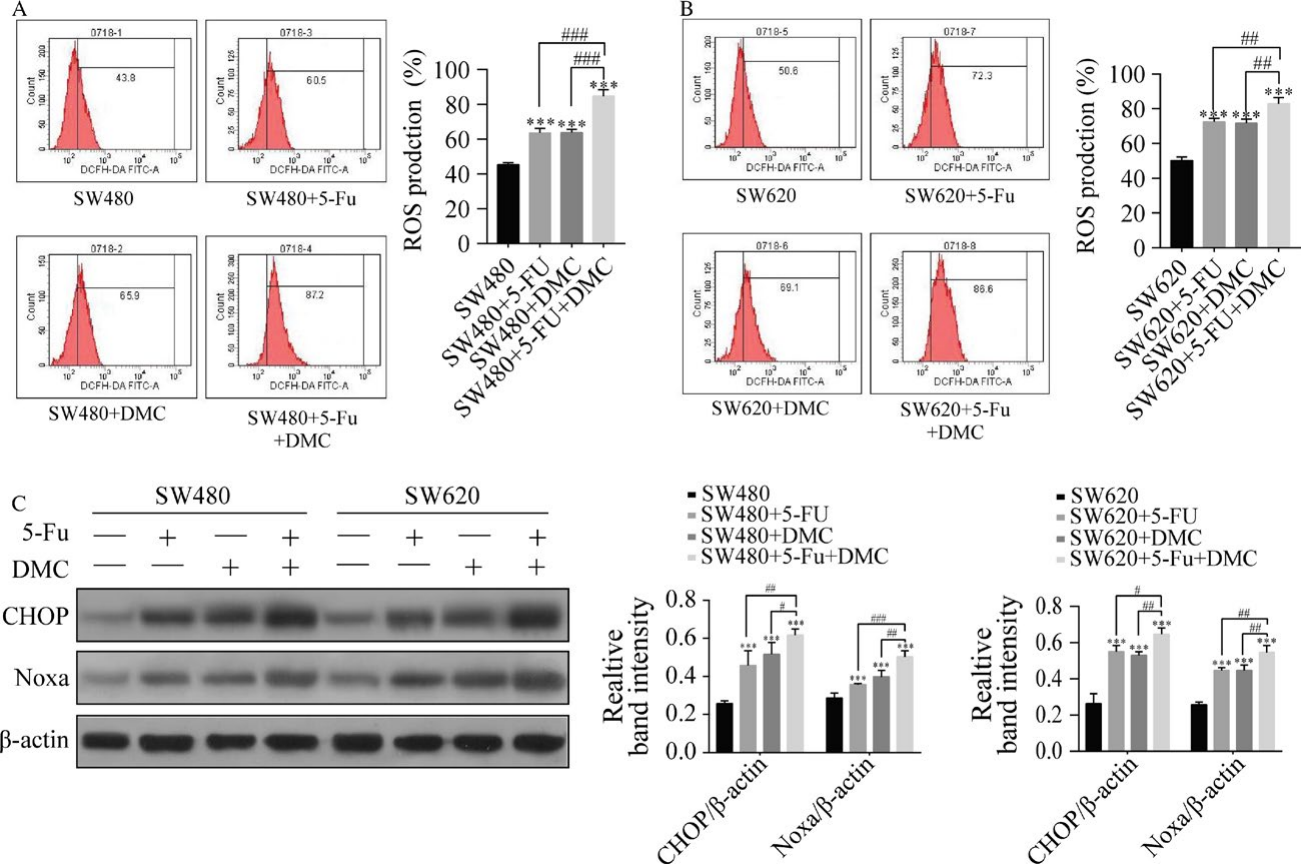
The effects of 5‐Fu and/or DMC on reactive oxygen species (ROS) production and ROS pathway related protein expressions. The 2′,7′‐dichlorodihydrofluorescein diacetate assay was used for the detection of ROS in SW480 (A) and SW620 cells (B). ROS levels were significantly increased after DMA and 5‐Fu treatment in both cell lines. The absolute intensity values of CHOP and Noxa were significantly different between cell lines (C). Results were obtained from three independent experiments and expressed as the means SD. Compared with SW480 (or SW620), *P < 0.05, **P < 0.01, *P* < 0.001; compared with SW480 (or SW620) + 5‐Fu + DMC, ^#^ *P *< 0.05, ^##^ *P* < 0.01, ^###^ *P* < 0.001

ROS production was measured by flow cytometry. ROS production rates in SW480 and SW620 cells were significantly increased in group B (63.60 ± 2.70% and 72.47 ± 1.96%, respectively), C (63.83 ± 1.83% and 71.73 ± 2.37%, respectively), and D (84.73 ± 3.76% and 82.90 ± 3.34%, respectively) compared with group A (45.27 ± 1.31% and 50.17 ± 1.99%, respectively). ROS production was also significantly increased in group D compared with groups B and C. Protein expression of CHOP and Noxa were significantly increased by DMC and 5‐Fu treatments in both SW480 and SW620 cells, and expression was higher in group D compared with groups B and C. The results are shown as the representative experiment or the means ± SD of three experiments in Figure 5.

**Figure 6:**
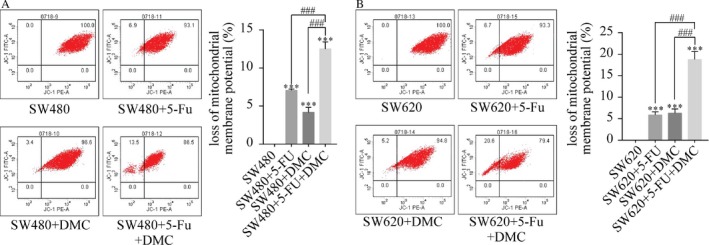
The effects of 5‐Fu and/or DMC on mitochondrial membrane potential. The proportion of cells with reduced mitochondrial membrane potential was significantly increased by DMC and 5‐Fu treatment in SW480 (A) and SW620 cells (B). Results were obtained from three independent experiments and expressed as the means ± SD. Compared with SW480 (or SW620), ***P < 0.001; compared with SW480 (or SW620) + 5‐Fu + DMC, ^###^ *P* < 0.001

The proportion of cells with decreased mitochondrial membrane potential was measured by flow cytometry. The proportion of SW480 and SW620 cells with decreased mitochondrial membrane potential was significantly increased in groups B (7.07 ± 0.15% and 5.87 ± 0.74%, respectively), C (4.17 ± 0.67% and 6.30 ± 0.95%, respectively), and D (12.50 ± 0.92% and 18.77 1.85%, respectively) compared with group A (0.00% and 0.00%, respectively). In addition, the proportion of cells with decreased mitochondrial membrane potential was increased in group D compared with groups B and C in both SW480 and SW620 cells. The results are shown as the representative experiment of the means ± SD of three experiments in Figure 6.

### Endoplasmic reticulum expansion

**Figure 7:**
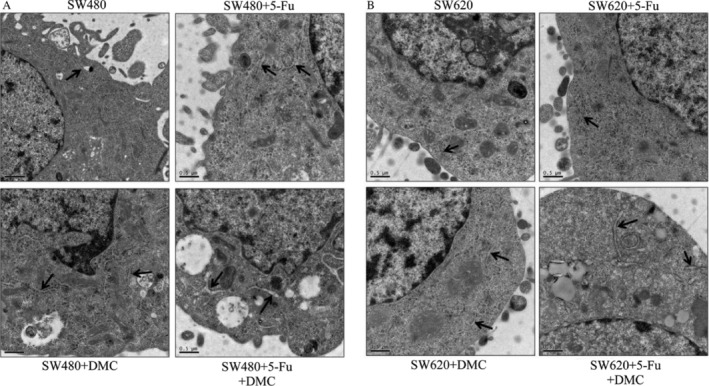
The effects of 5‐Fu and/or DMC on endoplasmic reticulum expansion. Endoplasmic reticulum expansion (black arrows) was significantly enhanced by DMC and 5‐Fu in SW480 (A) and SW620 cells (B).

Treating cells with DMC or 5‐Fu alone resulted in significant endoplasmic reticulum expansion in SW480 cells and slight expansion in SW620 cells. In group D, significant expansion was observed in both SW480 and SW620 cells as shown in Figure 7.

## Discussion

Curcumin exerts a number of antitumor properties by modulating different molecular regulators 20. Moreover, a variety of studies demonstrated that DMC is more effective than curcumin at inducing apoptosis and promoting pro‐oxidant activity 21, 22, 23. Nevertheless, few studies on the effects of DMC had been performed until recently. In breast carcinoma MCF7 cells, DMC induced cell death through apoptosis and S‐phase arrest 24 and triggered strong proteasomal inhibition and high induction of CHOP to achieve potent antitumor effects against malignant breast cancer cells 22. Moreover, the apoptosis induced by DMC was mediated by the production of ROS, release of mitochondrial Cyt c, and subsequent activation of caspase‐3 in human renal carcinoma Caki cells 23. To the best of our knowledge, the only studies of DMC effects on colon cancer have been reported by our lab 11, which demonstrated that DMC exerts an effective antitumor effect in colon cancer cells by downregulating survivin and upregulating E‐cadherin.

This study showed that both DMC and 5‐Fu significantly inhibited the growth of two colon cancer cell lines. Moreover, the combined antitumor effect of DMC and 5‐Fu was greater than the effect of either drug alone. The combination index calculated in this study indicated a combinatorial effect of the two drugs. The combinatorial effect of DMC and 5‐Fu in SW480 and SW620 cells was reported for the first time in this study, but other studies have shown similar synergistic effects in HT‐29 cells 25.

Apoptosis, ROS, cell cycle phase arrest, mitochondrial membrane potential, and endoplasmic reticulum expansion play key roles in tumor regulation. Moreover, the pathways controlling these processes can interact and regulate each other 26. In this study, we found that both DMC and 5‐Fu can induce apoptosis, stimulate G0/G1 phase arrest, increase ROS production, decrease mitochondrial membrane potential, and enhance endoplasmic reticulum expansion. In addition, treating cells with both DMC and 5‐Fu resulted in increased efficacy compared with either treatment alone. The increase in apoptosis was confirmed by upregulation of Bax and Cyt c and downregulation of Bcl2. The increase in ROS was confirmed by upregulation of CHOP and Noxa. These outcomes play key roles in the antitumor effects of DMC. Similarly, curcumin has been shown to induce apoptosis in vitro 27, 28 and in vivo 29, induce G0/G1 phase arrest 30, increase ROS production 26, 31, and decrease mitochondrial membrane potential 26 in colorectal cancer cells.

In conclusion, this study showed that DMC and 5‐Fu have an additive antitumor effect on colon cancer cells. In addition, the antitumor effect was closely related to the induction of apoptosis and G0/G1 phase arrest, increased ROS production and endoplasmic reticulum expansion, and decreased mitochondrial membrane potential.

## Conflict of Interest

None declared.
